# Bone marrow stem cells to destroy circulating HIV: a hypothetical therapeutic strategy

**DOI:** 10.1186/s40709-018-0075-5

**Published:** 2018-02-05

**Authors:** Umesh Chandra Halder

**Affiliations:** Department of Zoology, Raniganj Girls’ College, Searsole, Rajbari, Raniganj, Paschim Barddhaman, West Bengal 713358 India

**Keywords:** HIV, AIDS, HIV therapy, Hematopoietic stem cell, Red blood cell, HIV receptors, Burst-forming unit-erythrocyte

## Abstract

Human immunodeficiency virus (HIV) still poses enigmatic threats to human life. This virus has mastered in bypassing anti retroviral therapy leading to patients’ death. Circulating viruses are phenomenal for the disease outcome. This hypothesis proposes a therapeutic strategy utilizing receptor-integrated hematopoietic, erythroid and red blood cells. Here, HIV specific receptors trap circulating viruses that enter erythrocyte cytoplasm and form inactive integration complex. This model depicts easy, effective removal of circulating HIV without any adverse effect.

## Background

Having around 36 years of understanding after its discovery [1], scientists are still haunting a full and effective cure for human immunodeficiency virus (HIV) and its outcome acquired immunodeficiency syndrome (AIDS). Till date thousands of research efforts have revealed plethora of information regarding its life cycle [2–8] that characterized enigmatic AIDS. These immense knowledge provided possible targets for AIDS therapy [3–5, 8–11] that intervene entry, replication, packaging or budding of the virus leading to anti-retroviral therapy (ART) [12–17]. Apart from having diverse side-effects, ART has certain limitations too, as it only delays patients’ death but does not cure AIDS and also it only targets replicating HIVs and not the latent viral particles. Moreover, in doing so, it evokes successive immune compromising reactions making the situation worse. Circulating replicative HIV remains the biggest threat toward successful AIDS therapy. Therefore, an effective strategy is essential that can confer resistance towards circulating HIV particles. If replicating HIV particles were somehow eliminated, it would greatly reduce the effective viral burden from human body. On the other hand, latency provide base for long term existence of HIV without eliciting any immune response hiding deep inside immune organs [18–20]. Very recently, other than anti-retroviral drugs, such as experimentally promising HIV vaccine [21], neutralizing antibodies [22–24] and Clustered regularly interspaced short palindromic repeat-CRISPR-associated protein-9 nuclease (CRISPR-Cas 9) have shown effectiveness against HIV [25, 26] with certain limitations [27].

The most fascinating event in the viral life cycle is that only a few viral proteins effectively control and direct the cellular pathways for their own sake. So, knowledge of viral proteins functioning in the virus life cycle and properly targeting them may confer successful elimination of HIV from human body.

## Hypothesis

**Fig. 1:**
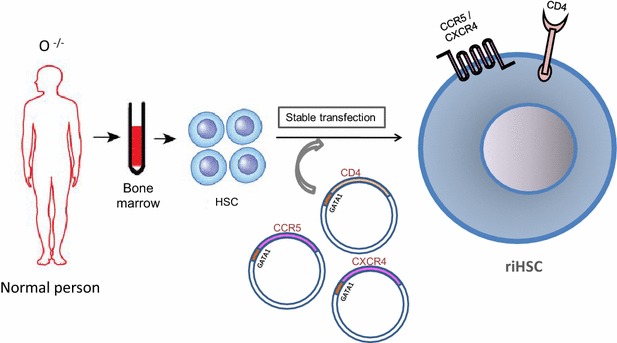
HIV receptor integrated hematopoietic stem cell formation. Hematopoietic stem cells can be collected from healthy person having O-negative (O^−/−^) blood group. HIV receptor-CD4 and co-receptors-CCR5 and CXCR4 will then be transgenically introduced onto the hematopoietic stem cell membranes by stably transducing or transfecting HSC with receptor genes under GATA-1 promoter control to form riHSC using viral or non-viral vectors. These receptor positive stem cells will be sorted using HIV receptors for next treatment. *HIV* human immunodeficiency virus, *HSC* hematopoietic stem cell, *CD4* cluster of differentiation 4, *CCR5* C-C chemokine receptor type 5, *CXCR4* C-X-C chemokine receptor type 4, *riHSC* receptor-integrated hematopoietic stem cell

**Fig. 2:**
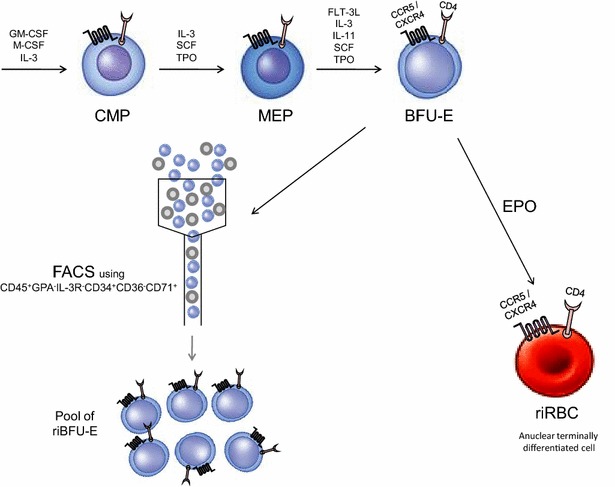
In vitro formation of riBFU-E and mature riRBC from riHSC. The formed riHSC from the stem cells will then be treated with several in vitro growth and differentiation factors like GM-CSF, M-CSF, IL-3, SCF, TPO etc. to obtain BFU-E having HIV receptor-co-receptor complex. Then, these riBFU-E cells will be selected and isolated from other cell lineages by FACS using CD45^+^GPA^−^IL-3R^−^CD34^+^CD36^−^CD71^+^ markers to finally yield pool of pure riBFU-E cells. Another large population of cells having mixed population of riBFU-E and other lineages like lymphoid and myeloid cells will be treated by EPO to finally obtain only mature riRBC. *GM-CSF* granulocytemacrophage colony stimulating factor, *M-CSF* macrophage colony-stimulating factor, *IL-3* interleukin-3, *IL-11* interleukin-11, *SCF* stem cell factor, *TPO* thrombopoietin, *FLT-3L* Fms-related tyrosine kinase 3 ligand, *CMP* common myeloid progenitor, *MEP* megakaryocyte/erythrocyte progenitor, *BFU-E* burst forming unit-erythroid, *riRBC* receptor integrated red blood corpuscle, *FACS* fluorescence-activated cell sorting, *riBFU-E* receptor integrated burst forming unit-erythroid, *EPO* erythropoietin

Previously transgenic mice showed effectiveness against Coxsackie virus B infection [28]. Here in this hypothesis, a therapeutic strategy has been proposed for AIDS treatment that would utilize bone marrow stem cells. The proposed therapeutic strategy exploits receptor-integrated red blood corpuscles (riRBC) to trap and finally kill the circulating HIVs. According to this model, RBC membranes can be loaded with cluster of differentiation 4 (CD4) receptor along with C-C chemokine receptor type 5 (CCR5) and C-X-C chemokine receptor type 4 (CXCR4) co-receptors [4, 6, 7] that will specifically bind circulating HIV particles. The whole process can be divided into four stages. Firstly, hematopoietic stem cells (HSC) can be collected from bone marrow of O-negative (O^−/−^) healthy person to negate possible future immune reaction. Then, these cells will be stably transduced or transfected with CD4, CCR5 and CXCR4 genes using highly efficient viral (retroviral, lentiviral etc.) [29–31] or non-viral (episomal) based vectors [32–35], cloned from T_H_-lymphocyte genome, under the control of GATA-1 promoter [36] which can be sorted using over-expressed HIV-receptors (Fig. 1). Secondly, after sorting the in vitro receptor-integrated HSCs (riHSC) will be subjected to treatment with different growth and transformation factors (GM-CSF, M-CSF, IL-3, SCF, TPO, FLT-3L, IL-11) in the medium to finally obtain receptor-integrated burst-forming unit-erythrocyte (riBFU-E) stem cell [37–49]. Moderate quantities of riBFU-Es will then be selected from the cell pool by FACS using CD45^+^GPA^−^IL-3R^−^CD34^+^CD36^−^CD71^+^-markers [50, 51] and the rest will be treated in the culture medium using EPO (erythropoietin) [39, 43, 47] to yield huge pool of mature erythrocytes or riRBC having membrane integrated CD4, CCR5 and CXCR4 (Fig. 2). Along with the growth factors, certain rejuvenating factors and strategies [52–55] will be employed throughout the in vitro process to curb stem cell aging which is an evident stem cell therapeutic problem [56]. Third step will employ a combination therapy by transfusing riRBCs along with the riBFU-E stem cells in the peripheral blood of AIDS patient.

**Fig. 3:**
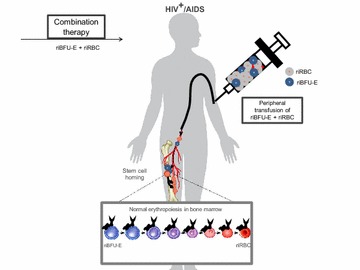
Combination therapy to transfuse riBFU-E and riRBC into HIV-positive patient. riBFU-E and riRBCs will be transfused into peripheral blood of HIV patients as combination therapeutic strategy. Once inside patients’ body, riRBCs will be immediately functional to counter circulating HIV particles. On the other hand, riBFU-E from the peripheral blood will find its way to the bone marrow by stem cell homing and will normally differentiate into riRBC that will further amplify the efficiency of the therapy. *riRBCs* receptor integrated red blood corpuscles, *riBFU-E* receptor integrated burst forming unit-erythroid

**Fig. 4:**
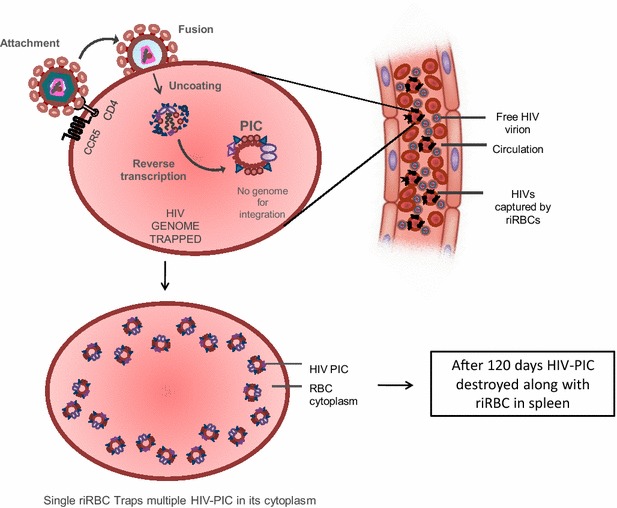
Trapping HIVs within riRBC and destroying them. Once in blood, the riRBCs will engage to bind free HIV particles by the interaction of viral glycoproteins gp 120 and gp 41 with its membrane embedded CD4, CCR5 and CXCR4. After attachment fusion of viral and riRBC membrane will result in uncoating of viral core contents within cell cytoplasm leading to reverse transcription using its own transcriptase to form PIC. A single riRBC will be able to trap numerous HIV-PIC. However, once PIC formed it will remain inert as it will hardly find any genome to integrate and finally will be destroyed along with aged riRBC in spleen. *HIVs* human immunodeficiency viruses, *CD4* cluster of differentiation 4, *CCR5* C-C chemokine receptor type 5, *riRBC* receptor integrated red blood corpuscle, *PIC* pre integration complex

Soon after transfusion the riBFU-Es will find its way to the bone marrow by ‘stem-cell homing’ [57–60] and once in the specific niche the engineered stem cell will proliferate to yield numerous riRBCs naturally from the bone marrow (Fig. 3). Bone marrow derived riRBCs along with the transfused riRBCs in the peripheral blood will then engage in receptor mediated capturing of circulating HIV particles. The fascinating feature of this engineered riRBC is that a single riRBC can confine numerous HIV particles till its natural destruction after 120 days in patient’s body. Once circulating HIVs bind with the receptor and co-receptor associated complex on the riRBC membrane by its envelop glycoproteins gp 120 and gp 41, viral membrane will readily fuse with cell membrane leading to partial core shell uncoating and entry into riRBC cytoplasm [4, 6]. This event would immediately facilitate reverse transcription of genomic HIV-RNA to yield pre-integration complex (PIC) [4, 6, 11, 61]. Up to this event HIV follows its normal path of infection but once PIC has been formed inside riRBC cytoplasm, due to the lack of nucleus it will be unable to complete its replication and life cycle and eventually will stall. Single riRBC will carry numerous of such PICs and eventually be destroyed along with the riRBC by the action of macrophagic lytic enzymes in the spleen [62]. According to this hypothesis the proposed therapeutic strategy will greatly reduce the HIV burden from the AIDS patient abruptly, and effectively (Fig. 4).

## Conclusions

The credentials of this hypothetical therapeutic strategy will be immense since (i) as the riHSC is a stem cell, once transduced cells are generated they will serve to produce riBFU-E and riRBC for a long period of time, (ii) riBFU-E as a stem cell will proliferate in the bone marrow for a long period of time to produce numerous riRBC naturally without forming lymphoid lineages of cells (modern improved techniques permit successful, efficient and enormous production of RBCs from HSCs [37–49] and this will enable successful large scale production of riRBCs), (iii) both the riBFU-E and riRBC cells will not elicit any immune reaction as the cells are immunologically non-reactive taken from healthy O^−/−^ person and (iv) the HIV trapping riRBCs will remain effective for around 120 days to continuously carry out its function to entrap circulating viruses. Moreover, early findings of HIV binding to RBCs have been reported that remain bound by Duffy antigen receptor (DARC) and spread infection to T-cells [63–66]. These findings further strengthen the present method as in presence of overexpressed HIV binding receptors on riRBC, HIV particles will readily enter the cell for their dead end. Another concern may be with the proper alignment of overexpressed receptors facilitating HIV binding. But as T-cells derive from lymphoid lineage from HSCs, hence, it is expected that overexpressing CD4, CCR5 and CXCR4 receptor genes in HSCs will yield homologous receptor like on T cell for HIV binding under specific growth and differentiation factor influence.

Safety is a big issue in genetically modified therapy so, proper safety measures and precautions should be taken [67–70] and whole procedure must be critically followed to prevent any accidental transmission of modified stem cells. Finally, it should be noted that effective strategies must combine this one to cope up inactive hidden latent viruses to completely remove HIV from human body and these strategic riRBCs not only serve as a vehicle to fight HIV but little modification will enable it to battle numerous other infections too.

## Abbreviations

HIV: human immunodeficiency virus
HSC: hematopoietic stem cell
CD4: cluster of differentiation 4
CCR5: C-C chemokine receptor type 5
CXCR4: C-X-C chemokine receptor type 4
riHSC: receptor-integrated hematopoietic stem cell
GM-CSF: granulocytemacrophage colony stimulating factor
M-CSF: macrophage colony-stimulating factor
IL-3: interleukin-3
IL-11: interleukin-11
SCF: stem cell factor
TPO: thrombopoietin
FLT-3L: Fms-related tyrosine kinase 3 ligand
CMP: common myeloid progenitor
MEP: megakaryocyte/erythrocyte progenitor
BFU-E: burst forming unit-erythroid
riRBC: receptor integrated red blood corpuscle
FACS: fluorescence-activated cell sorting
riBFU-E: receptor integrated burst forming unit-erythroid
EPO: erythropoietin
riRBCs: receptor integrated red blood corpuscles
riBFU-E: receptor integrated burst forming unit-erythroid
HIVs: human immunodeficiency viruses
CD4: cluster of differentiation 4
CCR5: C-C chemokine receptor type 5
riRBC: receptor integrated red blood corpuscle
PIC: pre integration complex
DARC: Duffy antigen receptor
